# Categorization of Food Consumption Patterns in Indigenous Communities of the Quilotoa in Ecuador

**DOI:** 10.1002/fsn3.4717

**Published:** 2025-02-03

**Authors:** Edgar Wilson Rojas, Sofía Benítez, Myriam Jicela Andrade, Luis Castillo, Rosmerie Ochsner, Nelly Sarmiento

**Affiliations:** ^1^ Nursing Faculty, Nutrition and Dietetics Unit Pontificia Universidad Católica del Ecuador Quito Ecuador; ^2^ Science Faculty Universidad Central del Ecuador Quito Ecuador

**Keywords:** food consumption patterns, food groups, frequency of food consumption, high‐calorie foods, Indigenous farmers

## Abstract

Preliminary evidence suggests that rural areas have poor nutritional indicators despite their self‐sufficient local production. Thus, this study aimed to categorize the food consumption patterns of the rural Indigenous population next to the Quilotoa Lagoon in Ecuador based on the frequency of food intake. Data were obtained from 258 Indigenous farmers using structured and validated consumption frequency questionnaires. The consumption of 91 foods classified into eight groups was evaluated using concordance indicators, correlation analysis, and multivariate techniques such as principal component analysis and correspondence factor analysis. Consumption levels were categorized by stratifying the proportion of families that consumed each food item. Four consumption pattern types were identified. The category with “high consumption” foods, greater than 66.8%, was based on foods rich in carbohydrates, such as rice (89.3%), potatoes (88.9%), oats (74.1%), and *morocho* (74%). Protein intake was limited to eggs (82%) and fish (73.5%). Vegetables, such as carrots (90.4%) and onions (96.1%), were highly consumed but classified as condiments in meal preparation. Chicken (66.5%) and milk (61%) were categorized as “moderate consumption.” Local foods such as *melloco* (37.1%) and oca (28.2%) were classified as “low consumption”. Lastly, the most produced local food, chocho, had a consumption rate of 14.3%, which was considered “very low consumption”. When analyzing consumption patterns by area, the Chugchilan parish with High Center or Quilotoa‐Paved Road zones showed very good concordance (0.81 and 0.79, respectively), while the Subtropical zone had less concordance (0.73). Foods rich in high‐calorie carbohydrates were the most commonly consumed across all four consumption patterns.

## Graphical abstract

Four consumption pattern types were defined. The category “high consumption” includes carbohydrates such as rice (89.3%), potatoes (88.9%), oats (74.1%), and *morocho* (74%). Protein intake was limited to eggs (82%) and fish (73.5%). Vegetables such as carrots (90.4%) and onions (96.1%) were classified as condiments. Chicken (66.5%) and milk (61%) were categorized as “moderate consumption.” Local foods, including *melloco* (37.1%) and oca (28.2%), were classified as “low consumption,” while the chocho (14.3%) was classified as “very low consumption.”

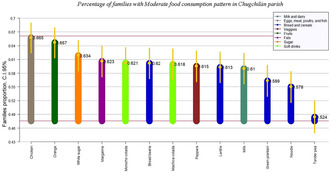

## 1 Introduction

Assessing the nutritional status of a population involves analyzing the quality and quantity of food consumed, allowing for the diets of individuals and communities to be characterized (Ramírez et al. 2017). Food consumption patterns, assumed to be synonymous with food patterns, are understood as the frequency with which a person eats different foods or food groups to meet nutritional and social needs, depending on their habits, customs, and place of residence (Caballero Gutiérrez 2017; Ekmeiro Salvador et al. 2015). This definition of food patterns is related to community food practices and how food is acquired by a family and/or population, which, in turn, is determined by the market, availability, economic income, and product costs (Duana Ávila 2004; Ramírez et al. 2017). In addition, consumption levels (proportion of families that consume food) are related to families' acquisition of products in the market once a week (Sánchez 1985).

To understand food consumption in a community, there is a set of techniques and procedures regarding food supply, storage, preparation, and consumption (Couceiro 2007). This consumption reveals the food culture of a human group in its interrelation with macro‐ and micro‐social factors, such as the incorporation of new foods and their adaptation to daily life in a society marked by ideals of progress and development (Appadurai 2001). Food consumption is the aggregate of habits that ensure adequate nutrition at different stages of life, involving the beliefs, customs, politics, religion, culture, environment, education, and psychological factors of an individual (Barrial and Barrial 2011).

Food consumption in México and Latin America has shown that the population of the rural highlands has one of the lowest levels of protein consumption and a high consumption of carbohydrates such as rice, sugar, and white bread (Morón and Schjtman 1997; Shamah Levy et al. 2014). However, the level of consumption of other foods that contains nutrients, such as iron, calcium, zinc, and Vitamin A, is still unknown, indicating low dietary quality (Villacreses et al. 2017). Deficits in these micronutrients negatively impact the neurological, physical, and mental development of individuals, particularly in children under 5 years old (Freire et al. 2014; Villacreses et al. 2017).

In the rural highlands of Ecuador, 38.4% of children under 5 years old suffer from chronic malnutrition, and the prevalence of overweight and obesity was 32.6% (INEC 2018). Cotopaxi is one of the provinces with the highest rates of chronic child malnutrition (CCM) among children under 5 years, affecting 29.5% of infants. Within this province, Chugchilán is located in the canton of Sigchos, next to Quilotoa Lagoon. At the end of 2015, the GADM Sigchos registered 43% of families in this town with severe food insecurity (GADM Sigchos 2015), while the INEC in 2024 reported a very high CCM rate of 44.6% for children under 5 years (INEC 2024). This issue is particularly concerning given that the dietary causes of malnutrition among remote populations, such as the Indigenous communities in the Ecuadorian highlands, are often unknown. Food security challenges arise from limited access to nutritious food, especially for low‐income families (GAD Cotopaxi 2015).

The local production of this Andean community is supposed to be for self‐sufficiency, but studies need to analyze their food consumption patterns to explain their nutritional problems. In this context, the Pontificia Universidad Católica del Ecuador is developing a research project titled “The use of pesticides in family/community agriculture and its influence on the quality of food consumed by and the health of Indigenous populations.” One of the objectives is to describe the food consumption patterns of the Andean Indigenous communities' diets in the central highlands of Ecuador. Using nonparametric estimations, categories of Andean Indigenous food patterns in the central highlands of Ecuador were proposed.

## 2 Materials and Methods

### 2.1 Study Design

This quantitative study used a cross‐sectional design to address the research question: What are the food consumption pattern characteristics of the rural Indigenous population by area of residence?

### 2.2 Study Population and Recruitment

The study population was from the township of Chugchilán in the Ecuadorian highlands. Most were originally *Kychwa* and *Panzaleo* Indigenous, and their main occupation was agriculture (GAD Chugchilán 2015; GADM Sigchos 2015). The Parish Council of Chugchilán and the National Institute of Statistics and Census reported that 1300 family farmers have lived in this area for the last 10 years. Based on the resident lists provided by the leader of each community, 258 households that practiced agriculture in the Chugchilán communities were randomly selected. We invited individuals over 18 years of age, who were responsible for the household food preparation, to participate.

### 2.3 Sample Size

The sample size calculation was performed using the n4Studies application and the proportion estimation calculation for a finite population. An adjustment was necessary to account for the loss of some research participants, mainly because of incomplete information or refusal to complete the questionnaire.

Based on the information obtained from the Parish Council of Chugchilán and the National Institute of Statistics and Census, we considered a sampling frame where *N* is the number of families living in the township of Chugchilán (1300); *Z* is the quantile of a normal distribution corresponding to 95% confidence; *p* is the frequency of the problem, which in the absence of previous information, is assumed to generate the most significant possible sample: 0.5; and *d* is the maximum estimation error, which in this case is 0.53 (adjusted for losses at the time of data collection). The final sample size was 271 families.

For this calculation, we considered weighted data, meaning that each household was weighted proportionally to the number of interviews conducted in the locality (Table 1).

**TABLE 1:** Distribution of households in the sample and population by area of residence.

Area of residence	Sample		Population	
	Households	%	Households	%
Quilotoa‐Paved Road	105	40.7	459	35.3
High Center	100	38.8	419	32.2
Subtropic	53	20.5	422	32.5
Total	258	100.0	1300	100.0

For comparison purposes, the information was disaggregated by geographic area of residence, considering three localities: Quilotoa‐Paved Road, High Center, and Subtropics.

### 2.4 Data Collection Techniques and Instruments

Fieldwork was conducted through home visits, which lasted an average of 1.5 h per family. Senior students of nutrition and nursing conducted these visits, and medical students, accompanied and supervised by research team members, covered 29 communities over 90 days.

A food pattern shows food consumption practices, and for this study, the consumption pattern was defined by Juarez (2001, as cited in Ávila 2004) as the consumption of set foods at least once a week, and it is remembered at least 24 h after their intake.

A semi‐quantitative food frequency questionnaire was developed and adapted from Pérez Izquierdo, Nazar Beutelspacher, Pérez‐Gil Romo, et al. (2012), Pérez Izquierdo, Nazar Beutelspacher, Salvatierra Izaba, et al. (2012) and distributed to the study participants during the visits. Based on previous information obtained from key community leaders, a list of 91 foods consumed by the families was created. This instrument allowed the collection of dietary information about the preferences, frequency, and servings of foods and beverages consumed by the study families during the last 3 months.

These and other instruments used in the project were previously validated linguistically in randomly selected families from communities with similar characteristics. The Research Department and Bioethics Committee of the Pontificia Universidad Católica del Ecuador approved the study protocol and data collection instruments and assigned the Code 2019‐73‐EO. All participants provided written informed consent. Before administering the surveys, students received training on how to apply data collection instruments, home visits, and data processing techniques.

The collected information was evaluated to verify its completeness and quality. Thirteen incomplete surveys were eliminated, resulting in 258 valid responses. Subsequently, the information was tabulated in Microsoft Excel spreadsheets, and once the databases were prepared, they were cleaned again to ensure quality.

### 2.5 Outcomes of Interest

The unit of analysis was the proportion of households that consumed each food established in the food frequency questionnaire. Despite a high response rate from the families, not all households responded to all the foods surveyed; therefore, these were not considered when making the estimates.

Swindale and Bilinksy (2006) explain that to more accurately reflect a quality diet, it is essential to assess it by food groups rather than merely counting the number of foods consumed. In this study, the 91 foods identified in the survey were categorized into eight groups based on the classification by (Serra and Aranceta 2006), as illustrated in Table 2. This categorization aimed to evaluate whether the dietary diversity criterion was satisfied within the consumption patterns, indicating a sufficient variety of macronutrients and micronutrients.

**TABLE 2:** Number of foods by food group.

Group	Number of foods	%
Bread and cereals	28	30.8
Veggies	15	16.5
Soft drinks	13	14.3
Eggs, meat, poultry, and fish	12	13.2
Fruits	10	11.0
Fats	5	5.5
Sugar	4	4.4
Milk and dairy	4	4.4
Total	91	100.0

### 2.6 Data Analysis

Data processing and statistical analyses were performed using the free software R Version 4.3.3. Initially, we structured food consumption patterns to determine the percentage of families that consumed each food. To this end, we developed a list of 91 foods consumed by the families. Next, we constructed food groups based on consumption levels. Then, we established the consumption patterns and relationships between the areas of residence using concordance indicators, correlation analysis, and multivariate techniques such as principal component analysis and correspondence factor analysis.

### 2.7 Determination of Consumption Patterns (Levels)

We used the square root accumulation for the strata technique proposed by Cochran (1990). This probability law governs the variable: the proportion of families that consume each food. Starting from the inflection points (where the density changes direction), some differences were perceived in the probability distribution according to the area; however, for comparison purposes, the cutoff points were considered from the estimated probability of the total sample.

**FIGURE 1:**
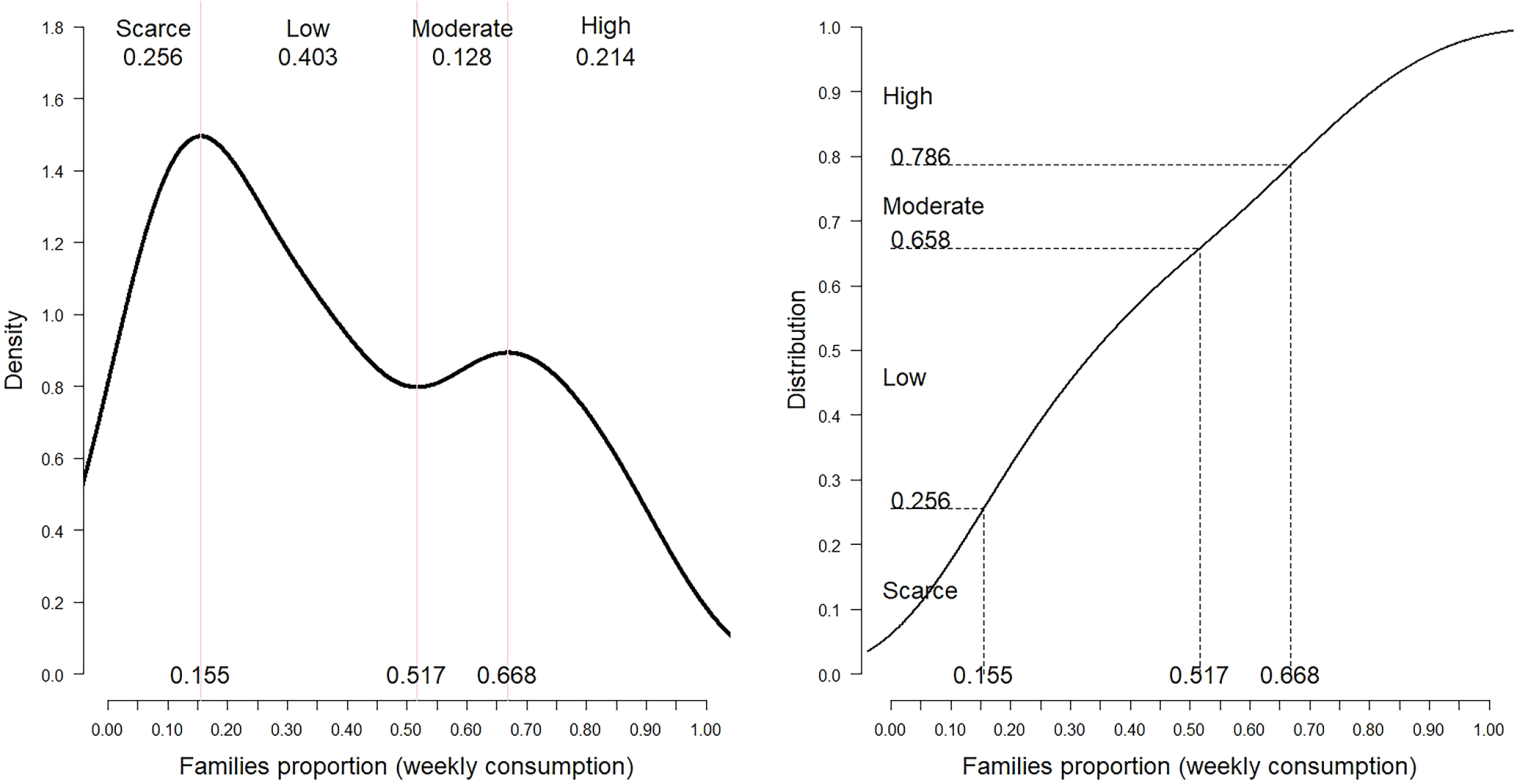
Inflection points (weekly consumption ranges).

This strategy marks the values 0.155, 0.517, and 0.668 as cut‐off points for consumption levels and categorizes the food consumption patterns as low, moderate, and high (Figure 1).

This categorization of food consumption patterns was supported by similar proposals, such as those by Kramis Joublanc (1994). Using the observed data, we obtained the distribution of food consumption patterns (Table 3).

**TABLE 3:** Distribution of food consumption patterns in the Chugchilán communities.

Consumption pattern	Expected range of consumption (%)^a^		% Range of food expected	Number of foods and percentage observed by rank							
				Total		QVP		CA		St	
				*N*	%	*N*	%	*N*	%	*N*	%
Null	0		0							5	5.5
Very low consumption	0	15.5	25.6	25	27.5	22	24.2	28	30.8	20	22.0
Low consumption	15.6	51.7	40.3	35	38.5	36	39.6	33	36.3	31	34.1
Moderate consumption	51.8	66.8	12.8	13	14.3	17	18.7	11	12.1	9	9.9
High consumption	66.9	100	21.4	18	19.8	16	17.6	19	20.9	26	28.6

Abbreviations: CA, High Center; QVP, Quilotoa‐Paved Road; St, Subtropic.

^a^ Expected range of percentage of households consuming (total).

Finally, we analyzed the similarities of consumption patterns of the parish farmers with the particularities of the patterns in their areas of residence. We considered possible differences in the patterns related to the ecological levels, differences in food production and availability, and some cultural differences between the Andean and Subtropic areas. Concordance analyses were performed to analyze these similarities. We used the quantitative interpretation of the kappa index to interpret the values obtained, where the maximum concordance was equal to 1 (*K* = 1) and concordance equal to 0 (*K* = 0) was caused by chance (Cavada Ch 2013).

Thus, the concordance levels were established as poor if the concordance was lower than 0.20, weak for values between 0.21 and 0.40, moderate for values between 0.41 and 0.60, good if the values were between 0.61 and 0.80, and very good for concordance values between 0.81 and 1 (Cavada Ch 2013; Cortés‐Reyes, Rubio‐Romero, and Gaitán‐Duarte 2010).

## 3 Results

**FIGURE 2:**
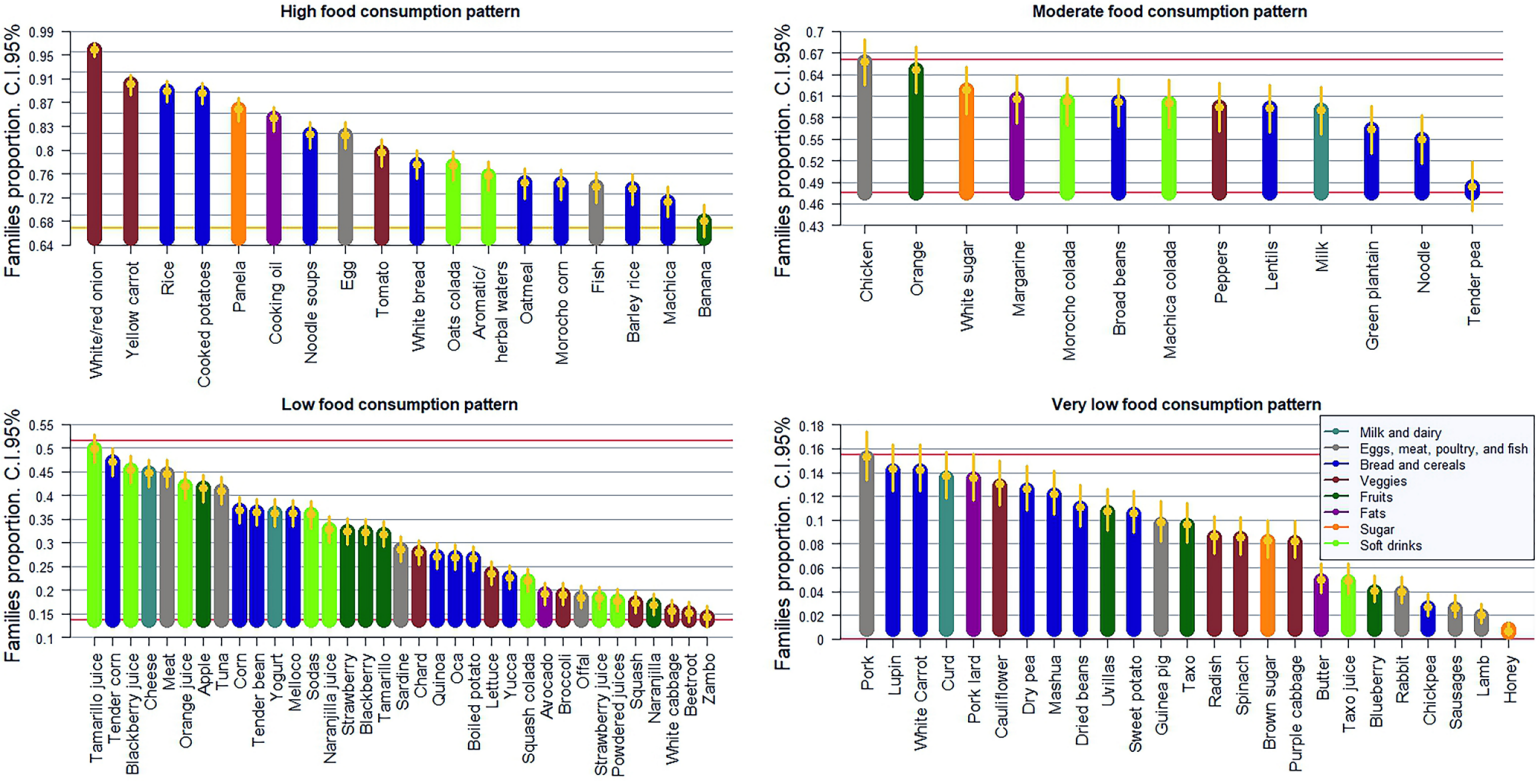
Percentage of families with high, moderate, low, and very low food consumption patterns in the Chugchilán parish.

Each food consumption pattern in the sample population, predominantly Indigenous farmers, was analyzed. We considered criteria such as food group diversity, Andean origin, agroproductive identity, and the importance of healthy eating by identifying the macro and micronutrient composition (Basulto et al. 2013). A food consumption pattern analysis was performed for the entire sample. This was disaggregated by geographic area of residence, which could reflect differences in consumption patterns influenced by cultural aspects and food availability (HLPE 2017). Figure 2 shows the eating patterns of each food and by food group that are consumed in Chugchilán.

When examining high biological value proteins, the consumption of eggs (82%) and fish (73%) is predominant, followed by chicken (66%). In contrast, meat (45%), pork (13%), and guinea pig (9%) are the least consumed by families.

Regarding dairy products, milk (61%) is consumed at a moderate level, while cheese (45%) and yogurt (37%) see lower consumption rates. Families indicate that the animals they raise and their products are primarily intended for sale or special occasions.

Foods rich in carbohydrates that dominate the community's consumption pattern primarily include those purchased from stores, such as rice (89%), noodle soup (82%), and white bread (77%). Additionally, there is a high to moderate consumption of cooked potatoes (88%), *morocho* corn (74%), barley rice (73%), and green plantain (58%). In contrast, foods like tender corn (47%), corn (37%), *melloco* (37%), quinoa (28%), oca (28%), yucca (24%), white carrot (14%), and mashua (12%) are consumed at low to very low levels, even though they are crops that could potentially be cultivated in the area.

In terms of fats, 84% of the women who cook report using vegetable oil, primarily palm oil, due to its affordability and efficiency.

It is important to emphasize that legumes, a valuable source of plant protein, are consumed at moderate to very low rates. For instance, broad beans account for 62%, lentils 61%, lupins 14%, while dry peas and chickpeas represent only 2%. This limited variety and low consumption of plant protein fail to sufficiently address the inadequate intake of foods rich in high biological value protein. Consequently, this may contribute significantly to the chronic malnutrition affecting the region (FAO 2015).

In the veggies category, there is a high consumption of white and red onions (96%), followed by yellow carrots (90%) and tomatoes (79%). Peppers are consumed at a moderate rate (61%), primarily used for seasoning, which means they often fall short of the recommended portion per person. The study reveals a low to very low consumption of other vegetables, such as chard (29%), lettuce (24%), cauliflower, radish, and spinach, with fewer than 13% of families including them in their diets.

When it comes to fruits, 68% of families consume bananas, and 65% consume oranges. Additionally, 42% have a moderate intake of apples, while 33% consume blackberries and 18% eat naranjilla. Other fruits with very low consumption rates include *uvillas* (10%) and *taxo* (9%). This limited intake of vitamins and minerals may contribute to nutritional imbalances that could negatively impact the studied population.

In Ecuador, the cultural significance of coladas is notable, particularly in Chugchilán, where consumption patterns include oats colada (77%), *morocho* colada (62%), and *máchica* colada (61%), along with herbal teas and infusions (75%). In terms of low and very low consumption, tamarillo juice (50%), blackberry juice (45%), orange juice (42%), sodas (36%), and *taxo* juice (4%) are observed. Notably, 86% of the families studied typically sweeten these beverages with panela.

### 3.1 Contrast of Food Groups and Consumption Patterns at the Parish Level

**FIGURE 3:**
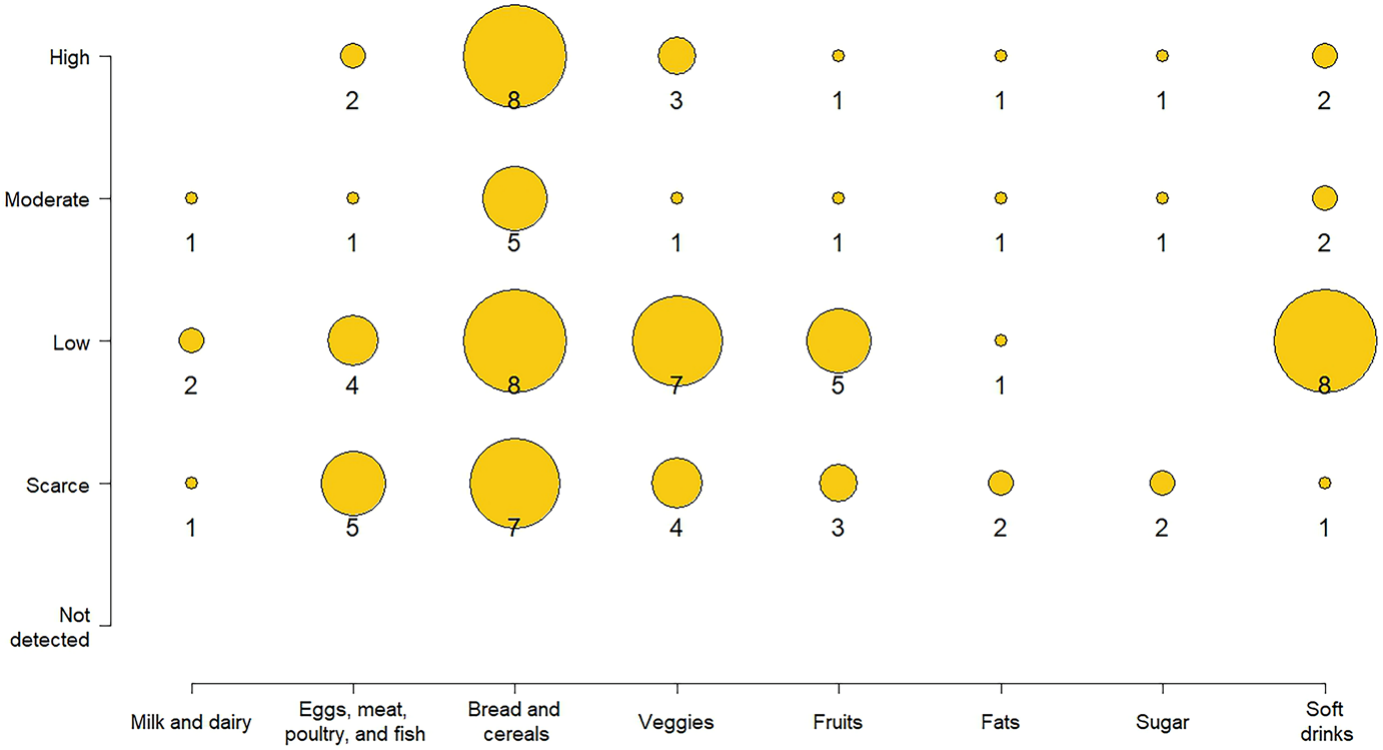
Distribution of the food groups and consumption patterns at the parish level.

Figure 3 shows the distribution of foods in the different groups for each food consumption pattern. The high‐carbohydrate bread and cereal group shows great homogeneity and contains some vegetable proteins, and its distribution varies between five and eight foods per category. The beverage group showed great heterogeneity, with a concentration of beverages (8) in the low consumption category. The veggies and fruit groups showed a slight difference, with some accumulation in the low and very low consumption categories, although a surprising number of vegetables correspond to high consumption (3). Despite providing essential micronutrients, their consumption is not ideal because these vegetables are consumed as a seasoning for some dishes. The egg, meat, and fish groups had a greater presence in the low and very low consumption groups. Finally, dairy products, fats, and sugars were distributed homogeneously and in small quantities across different consumption categories.

### 3.2 Food Consumption Pattern by Area of Residence

**FIGURE 4:**
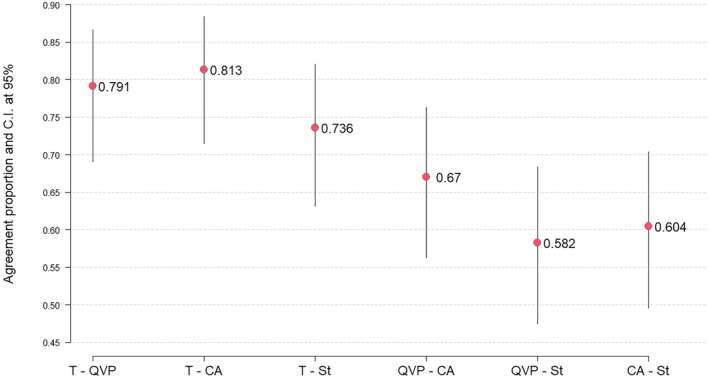
Concordance proportion of food consumption patterns by area of residence in the Chugchilán parish.

The concordance of food consumption patterns in the entire parish was compared with the patterns of Indigenous farmers in the three areas of residence (Figure 4).

When analyzing the concordance between zones, it is evident that Quilotoa‐Paved Road (0.79) and High Center (0.81) show greater alignment with the overall number of families in the parish compared to the Subtropics (0.73). A strong concordance is observed between Quilotoa‐Paved Road and High Center (0.67), which diminishes to moderate levels when comparing the Subtropics with High Center (0.60) and Quilotoa‐Paved Road (0.58). These variations in the consumption patterns of Chugchilán likely arise from their cultural roots, shaped by the predominant ethnicity in the region, as well as the diversity of ecological levels and the access routes for food distribution throughout the parish.

**FIGURE 5:**
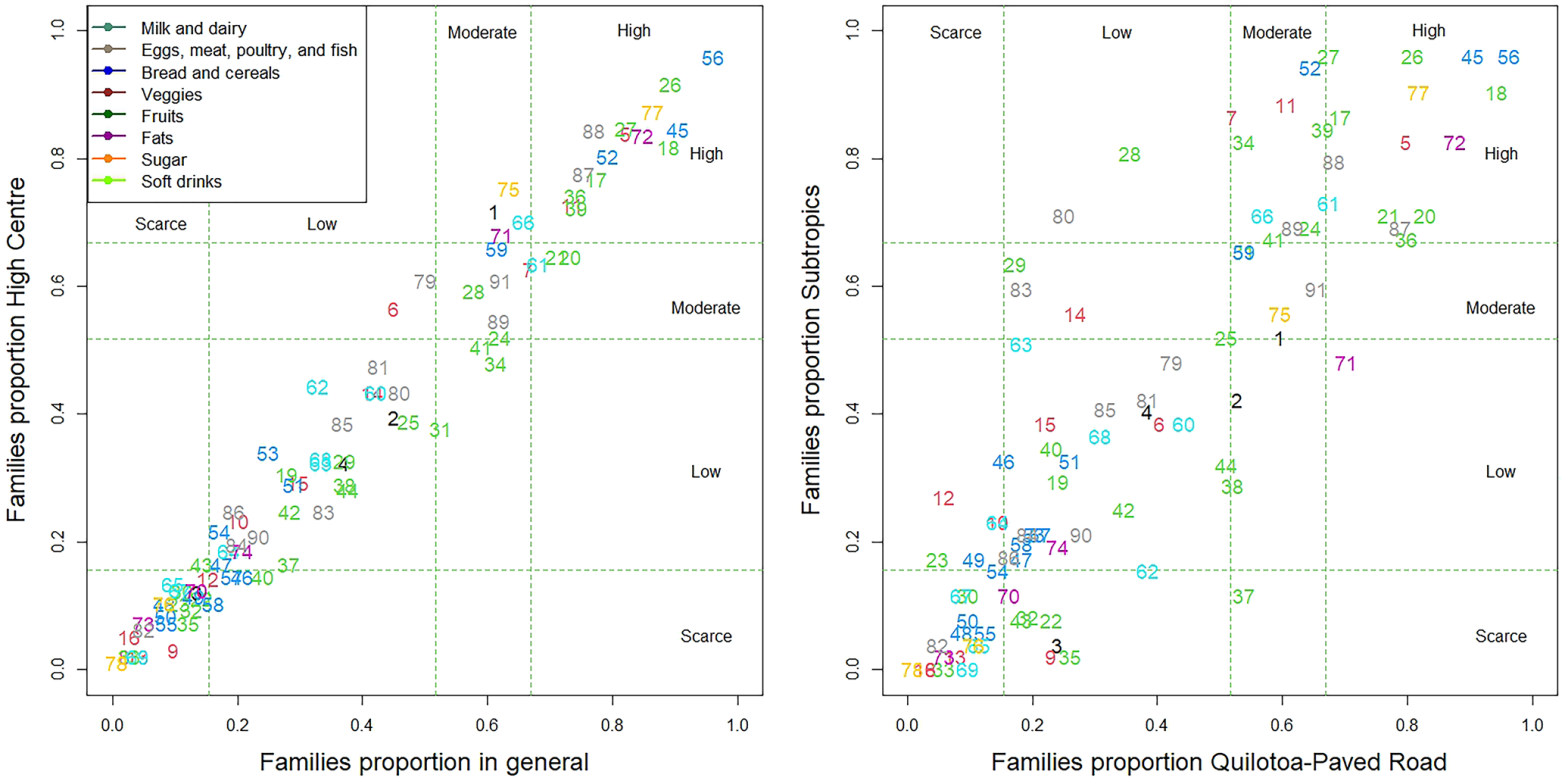
Concordance of food consumption patterns between families and area of residence in the Chugchilán parish.

In Figure 5, two graphs illustrate the concordance between different areas. When comparing the total number of families in the Chugchilán parish with those in the High Center, a positive linear dispersion is evident, indicating concordance across high, moderate, low, and scarce consumption levels. However, this pattern is not reflected in the graph comparing the Subtropical region with the High Center. In this case, there is a noticeable dispersion of foods toward the high and moderate consumption patterns of the Subtropical region, while the High Center exhibits the least food diversity.

The analysis reveals a significant concordance and a predominance of carbohydrates across the three zones. The most consumed foods include white bread (17), cooked potatoes (18), rice (26), noodle soup (27), *morocho* (36), oatmeal colada (88), panela (77), and palm vegetable oil (72). *colada de morocho* (91) is consumed at a moderate level. In terms of animal protein sources, there is little concordance, except for eggs (5). Among veggies, there is a notable consumption of white or red onions (56), along with moderate intake of bell peppers (59).

Foods with low to scarce consumption that show concordance across the three zones include quinoa (42), corn (44), white cabbage (47), Swiss chard (51), strawberries (68), avocado (74), *taxo* (65), *uvilla* (67), *mortiño* (69), and blackberry (63). Conversely, other foods with low to scarce consumption exhibit discordance among the zones.

Between Quilotoa‐Paved Road and High Center, there is a notable concordance in the moderate consumption of broad beans (24), chicken (7), and *colada de máchica* (89). Additionally, Quilotoa‐Paved Road and the Subtropical region share a high consumption pattern for barley rice (20) and *máchica* (21). There is also agreement between the Subtropical region and High Center regarding the high consumption of tomatoes (52).

For the following analysis, we will focus on elevated consumption patterns, as families report consuming these foods once a week, particularly protein sources and fruits, and up to three times a day for calorie‐dense foods. In the Subtropical region, there is significant consumption of green plantains (41), noodles (28), broad beans (24), lentils (34), oats (39), chicken (7), fish (11), banana (61), oranges (66), blackberry juice (80), and *colada de máchica* (89). In High Center, the foods with elevated consumption include oats (39), milk (1), fish (11), oranges (66), and white sugar (75). In the Quilotoa‐Paved Road area, banana (61) and veggetable shortening (71) are commonly consumed.

When categorizing foods, the Subtropical region features 15 items in the bread and cereals group, 4 in meats, 1 in dairy, 1 in veggies, 2 in fruits, 1 in fats, 1 in sugars, and 5 in soft drinks. In High Center, there are 9 foods in the bread and cereals group, 4 in meats, 1 in dairy, 2 in veggies, 1 in fruits, 2 in fats, 2 in sugars, and 4 in soft drinks. Finally, in the Quilotoa‐Paved Road area, the food distribution is as follows: 13 in bread and cereals, 3 in meats, 2 in dairy, 2 in veggies, 1 in fruits, 2 in fats, 1 in sugars, and 3 in soft drinks. The Subtropical region exhibits a greater variety of foods, likely due to its proximity to Maná, a city known for trade between the coast and the highlands.

## 4 Discussion

Eating is closely related to the sociocultural behavior of the population; this means that consumption patterns indicate what is usually consumed. However, some factors can disrupt a food pattern, and according to Caballero Gutiérrez (2017), they vary according to habits, customs, time, place, production, and food availability. In contrast, Ramírez et al. (2017) reported that the main factors are geographical and cultural. In Andean countries, dietary intake patterns are based on calorie‐dense cereal consumption, as in Ecuador, which maintains this dietary pattern of cereals and tubers (Morón and Schjtman 1997).

In the Chugchilán communities, Chanabá and Tobar (2017) analyzed the implementation of family gardens, and the highest percentage of food corresponds to carbohydrate food sources, which is reflected in the predominance of this type of food in the consumption patterns (Rojas et al. 2019). However, the type of starches that predominate in dietary patterns are refined and calorie‐dense, such as rice, bread, and pasta. Overweight and obesity, as well as cardiovascular diseases and diabetes mellitus, appear not only due to the high amount of carbohydrates consumed but also due to their quality. As they do not contain fiber, these foods have higher glycemic indices, increasing blood insulin requirements (Gordillo Cortaza et al. 2022). Likewise, there is no significant consumption of quinoa, *oca*, and *melloco*, which are native foods. This is probably because their consumption has decreased over the years owing to reduced cultivation, production, poor access to good‐quality seeds, marketing, and food choices, or their production is mainly destined for commercialization and little for self‐consumption (Anderson et al. 2016; Clavijo Ponce and Pérez Martínez 2014; Mazón et al. 2017).

A study conducted in the Bolivian Andes found that potatoes and quinoa are staple foods across the region, with quinoa recognized as a vital cereal for enhancing food security (Delgado and Delgado 2014). These findings align partially with the current study, which indicates that quinoa has low consumption in Chugchilán. This suggests a cultural loss, likely due to a diminishing interest in both consumption and production of quinoa. A similar decline has been noted in the consumption of fruits and vegetables, which are essential for various metabolic pathways (Caballero Gutiérrez 2017).

Regarding legumes such as lupin, lentil, chickpeas, or beans, their consumption is not significant for most families, even though this type of food has an important role in the parish's economy and is a valuable source of vegetable protein, fiber, vitamins, and minerals, especially in developing and emerging countries, where access to proteins of animal origin is often lacking and represents a serious nutritional issue (Lisciani et al. 2024) This may be due to the fact that the indigenous populations sell their crops to acquire other industrialized products, often influenced by the level of knowledge, publicity, and purchasing power (Morón and Schjtman 1997; Pérez Izquierdo, Nazar Beutelspacher, Pérez‐Gil Romo, et al. 2012; Pérez Izquierdo, Nazar Beutelspacher, Salvatierra Izaba, et al. 2012; Sánchez 1985), or simply to satisfy their hunger. According to a study in Colombia, low‐income families seek high‐calorie‐dense foods as a strategy for satiety and satisfaction (Arboleda, Duque, and Urrea 2013).

The consumption patterns of meat and dairy do not fulfill the daily protein requirements. While fish and eggs are included in the high consumption category, this does not necessarily mean they are consumed daily. Similarly, very few families consume dairy products, as well as beef, likely due to Chugchilán's geographical limitations; 81.56% of the area is covered by mountains and steep terrain, making it unsuitable for livestock farming. (GADP Chugchilán 2020). In contrast, guinea pigs are commonly raised in households, but only for family and religious events (Avilés et al. 2014).

The variety and consumption of fruits and vegetables in Chugchilán are alarmingly low, likely due to the inability of certain fruits and vegetables to adapt to the local soil conditions, which makes cultivation challenging (GADP Chugchilán 2020). This situation has a significant impact on the nutritional health of farming families, particularly children. The report titled “Social and Economic Impact of Malnutrition Carried out in Ecuador” emphasizes that malnutrition can result from both inadequate and excessive diet, leading to issues such as undernutrition, overweight, and obesity. The study found that a high intake of energy‐dense foods, combined with low consumption of protein and micronutrient‐rich food, greatly increases the risk of malnutrition problems (CEPAL et al. 2017).

Moreover, low‐income families often avoid purchasing micronutrient‐rich options like fruits and vegetables, resulting in a higher intake of calorie‐dense foods. This is frequently due to the lower caloric density of fruits and vegetables, which can make them appear more expensive (Arboleda, Duque, and Urrea 2013). These findings are consistent with a study conducted in the Department of Presidente Hayes (Paraguay), which revealed a high consumption of calorie‐rich foods and low intake of vegetables and dairy products, likely due to limited farming and the high cost of dairy products (Echagüe et al. 2015).

Rosique et al. (2010) conducted a study in Colombian indigenous communities, revealing that the consumption of protein, vitamins, and minerals was low in the dietary patterns of children under 5 years old, primarily due to food acculturation. This factor should be considered significant in understanding the chronic malnutrition observed in the communities of Chugchilán, where 23 cases of chronic malnutrition have been reported (GADP Chugchilán 2020). Those affected may experience stunted growth, diminished physical and mental development, and an increased susceptibility to diseases (Cilia, Rodríguez, and Aradillas 2015; UNICEF 2021).

When comparing the food groups and their consumption patterns identified in this study, carbohydrates emerged as the group with the greatest similarity. In terms of low and scarce consumption categories, the groups of veggies and fruits showed slight variations, as did the consumption of meat. Evidence indicates that animal protein sources include pork, guinea pig, rabbit, lamb, and livestock raised by families; however, consumption remains low due to high costs. These animals are primarily used for commercialization, reproduction, and the production of derived products, as well as for cultural, spiritual, and healing practices (De Zaldívar 2007; Morón and Schjtman 1997).

Additionally, there is a little variety of foods in the groups such as milk and dairy products, fats, and sugars, yet their distribution was relatively uniform. This aligns with findings from Larrea (2006), which confirmed that the dietary structure in the rural Sierra predominantly consists of foods high in carbohydrates but low in proteins, fats, and micronutrients.

When examining the concordance and its decline between the high zones of the parish and the Subtropics, it is noteworthy that, given the territory covers 32,250 ha (GADP Chugchilán 2020), significant changes in food consumption patterns should not be expected. However, in Ecuador, the two mountain ranges of the Andes create substantial slopes, enabling residents to traverse different ecological floors in short periods, thereby facilitating access to a variety of products (Auqui 2016; Moya 2010). This is particularly true in Chugchilán, which has an altitude range from 3970 to 618 m above sea level (GADP Chugchilán 2020). Consequently, the foods consumed in High Center and Quilotoa‐Paved Road differ from those in the Subtropics.

This decrease in concordance may also arise because High Center and Quilotoa‐Paved Road are situated in the highlands, where the population is predominantly indigenous (GADP Chugchilán 2020). In contrast, the Subtropical zone is influenced by its proximity to the canton of La Maná, which has a primarily *mestizo* and *montubio* population with customs and dietary patterns aligned with coastal regions (GAD Municipal La Maná 2015). According to GAD Cotopaxi (2015), the production that is prevalent in the highlands include corn, potato, and barley, while fruit production is concentrated in the Subtropical zone.

When examining food concordance across regions, consumption patterns evolve and adapt based on availability and necessity. The consumption of eggs is regarded positively, especially in families whose protein sources do not come from animal products (HLPE 2018). Barrial and Barrial (2011) suggest that the balanced diet should have sufficient macro‐ and micronutrients to meet the needs of the population. However, in all three regions, calorie‐dense foods dominate, with rice, white bread, and noodle soup leading the way. As the focus shifts toward calorie‐rich foods, there may be a displacement of fruits and vegetables, which are vital for growth and health. This occurs because calorie‐dense foods are often more accessible and convenient, leading to a reduced variety in the diet (IFAD 2021).

A study conducted in Carchi Province (northern Ecuadorian Andes) across three communities like those in Chugchilán revealed a predominance of carbohydrates, including potatoes, rice, sugar, noodles, and bread. In contrast, communities resembling the Subtropical region exhibited consumption patterns that included foods not typically found in highland areas, such as beans, bananas, and barley (Gross et al. 2015). Although the mentioned study does not focus on concordances, it highlighted that discordance may be based on altitude. This suggests that as elevation decreases, consumption patterns shift according to food availability and production.

In Chugchilán, agricultural production is influenced by the climatic conditions of the region, as certain crops may not thrive in the local soil or may yield poorly. For instance, *mortiño* (*Vaccinium floribundum*), which is endemic to the Ecuadorian moors (Coba et al. 2012), does not grow in the Subtropical zone.

Agricultural families often need to increase their intake of calorie‐rich foods to meet their energy demands, particularly in contexts involving intense physical labor. This necessity can lead to a preference for cereals and processed foods that are more calorie‐dense (FAO 2017). Additionally, they tend to favor the appealing flavors of processed foods, which significantly influences their lifestyle choices (Pérez Izquierdo, Nazar Beutelspacher, Pérez‐Gil Romo, et al. 2012; Pérez Izquierdo, Nazar Beutelspacher, Salvatierra Izaba, et al. 2012). Franco (2012) further notes that this preference is shaped by media influence, suggesting that life is perceived as easier with industrial products, often presenting foreign items as superior to local alternatives.

When examining the pattern of low and scarce consumption, there is consistency among the groups of fruits and vegetables, except for tomatoes, onions, and peppers, which are used almost daily to season dishes. Inadequate intake of micronutrients such as Vitamins A, D, and B, as well as iodine, zinc, magnesium, and iron, can lead to growth retardation, cognitive development delays, lethargy, rickets, low learning capacity, recurrent infections, malformations, visual deficiencies, anemia, and other noncommunicable diseases (Torres Mendoza, Morante Intriago, and Chilán Santana 2024).

Not benefiting from a nutrient‐diversified diet limits growth and developmental potential, as highlighted by UNICEF (2021). This limitation is evident in the height and learning outcomes of children in Chugchilán, where malnutrition is prevalent. Therefore, it is essential to consider not only the food groups, as noted by Swindale and Bilinksy (2006), but also the frequency of consumption, as well as the type and variety of foods within each group. However, analyzing food consumption by group indicates that families in all three areas do not consistently maintain a diverse dietary pattern, despite incorporating all eight food groups.

According to Caballero Gutiérrez (2017), Chugchilán is experiencing a nutritional transition characterized by a decline in the consumption of vegetables, fruits, juices, and stews, along with a decrease in high‐quality fats. Instead, there is an increased caloric intake from carbohydrates such as rice, noodles, pasta, and potatoes, as well as saturated fats primarily from processed and packaged foods (Pereira 2016; Sarmiento et al. 2015; Zapata, Rovirosa, and Carmuega 2016).

Cilia, Rodríguez, and Aradillas (2015) attribute this transition to factors such as migration, urbanization, economic development, and globalization, which are impacting Indigenous populations globally. These influences, leading to cultural erosion, become apparent through conversations with families and visits to local stores. Leyva Trinidad and Pérez Vázquez (2015) note that cultural food identity is shifting as the global market increasingly displaces local products with lower‐quality alternatives, thereby altering traditional eating habits and patterns.

This research offers valuable insights into how the environment, local traditions, and customs shape food diversity, providing a comprehensive understanding of trends and the predominant diet within consumption patterns. It also has significant social implications by identifying specific needs and opportunities for economic development, thereby promoting more sustainable and responsible local consumption through balanced and nutritious diets. Additionally, the research paves the way for integrating interdisciplinary approaches, which are essential for developing policies and programs tailored to the community's unique context.

However, the research may be influenced by biases in the information provided by some families about their eating habits, stemming from concerns about losing the solidarity bonus, which could compromise the accuracy of the data. Additionally, accessing remote communities could pose logistical challenges, affecting both data collection and participant engagement. Limitations in access to fresh and varied foods may not be fully captured in the data, which could impact the interpretation of the results. Moreover, insufficient funding and resources might restrict the research's scope, limiting the sample size and duration of the study. Lastly, the lack of relevant literature complicates the selection of appropriate methods for data collection and analysis, as well as hinders the comparison of results with similar studies, which could affect the overall quality of the critical reflection.

It is crucial to conduct complementary studies that delve deeper into the nutritional quality of the foods consumed and their preparations, thereby enhancing the connection between food and health. Additionally, the findings from this study should be shared with the communities of Chugchilán, enabling community leaders to implement interventions that raise awareness about the importance of a varied and balanced diet. This should focus on increasing the inclusion of fruits and vegetables, promoting the consumption of local products, and encouraging the diversification of crops.

## 5 Conclusions

When analyzing food consumption in Chugchilán, it is evident that all eight studied groups are represented. However, there is a notable predominance of foods in the bread and cereals category, which may overshadow other highly nutritious options found in the vegetable and fruit groups. While proteins such as eggs, fish, and chicken are consumed at a high to moderate level, this does not necessarily mean they are part of the daily diet. This indicates that the diet is primarily carbohydrate‐based, which is associated with malnutrition profiles and other health issues linked to this dietary behavior.

Moreover, the consumption patterns did not vary significantly across the three parish zones analyzed. However, a substantial difference is evident when comparing the Subtropical zone to the high Andean zones. The influence of the coastal region seems to enhance the diversity of foods consumed, highlighting the need for educational programs that emphasize the importance of a varied diet. It is also crucial to promote the consumption of locally grown foods over their sale, as well as to develop strategies that encourage the intake of fruits and vegetables while reducing reliance on processed foods with low nutritional value. This approach would particularly benefit children, improving their nutritional status and health, and enabling them to lead more competitive lives in the world.

## Author Contributions

**Edgar Wilson Rojas:** conceptualization (lead), data curation (equal), formal analysis (equal), funding acquisition (lead), investigation (equal), methodology (equal), project administration (equal), resources (lead), supervision (equal), validation (equal), visualization (equal), writing – original draft (equal), writing – review and editing (equal). **Sofía Benítez:** conceptualization (equal), data curation (supporting), formal analysis (equal), methodology (equal), supervision (equal), validation (equal), writing – original draft (equal), writing – review and editing (equal). **Myriam Jicela Andrade:** conceptualization (equal), formal analysis (supporting), investigation (equal), project administration (supporting), resources (supporting), supervision (equal), validation (equal), writing – original draft (equal), writing – review and editing (supporting). **Luis Castillo:** data curation (lead), formal analysis (equal), investigation (equal), visualization (equal), writing – original draft (equal), writing – review and editing (equal). **Rosmerie Ochsner:** formal analysis (equal), investigation (equal), supervision (equal), visualization (equal), writing – original draft (equal), writing – review and editing (equal). **Nelly Sarmiento:** formal analysis (equal), funding acquisition (supporting), investigation (equal), project administration (supporting), visualization (equal), writing – original draft (equal), writing – review and editing (supporting).

## Ethics Statement

The Ethics Committee for Research in Human Beings (CEISH) of the Pontificia Universidad Católica del Ecuador (PUCE), in the session of 04.11.2019, analized the project: “The use of pesticides in family/community agriculture and its influence on the quality of food and the health of Indigenous populations”. Once all the CEISH requirements were met, it was approved (Code 2019‐73‐EO) for an estimated duration of 1 year and 9 months. The project was implemented under the university's principles, philosophy, and mission, and was adjusted to the purposes and objectives of the PUCE guidelines and those indicated in the approved project. All the protocols were approved by the CEISH, including the informed consent and all the instruments applied in the research.

## Consent

The GOCIC‐CH is a partner in the research project; all the participants included in the study signed the informed consent.

## Conflicts of Interest

The authors declare no conflicts of interest.

## Data Availability

See https://www.dropbox.com/scl/fi/kjha9o91ivwdlpnizmz8d/BDD.xlsx?rlkey=7bjujih80peutz7blv0gj21lo&dl=0.
